# Iodine Requirements in Pediatrics: From Fetal Life to Adolescence

**DOI:** 10.3389/fendo.2022.929176

**Published:** 2022-07-01

**Authors:** Gabriella Iannuzzo, Angelo Campanozzi, Viola Trevisani, Irene Rutigliano, Veronica Abate, Domenico Rendina, Gianpaolo De Filippo

**Affiliations:** ^1^ Department of Clinical Medicine and Surgery, Federico II University, Naples, Italy; ^2^ Pediatrics, Department of Medical and Surgical Sciences, University of Foggia, Foggia, Italy; ^3^ Assistance Publique-Hôpitaux de Paris, Hôpital Robert-Debré, Service d’Endocrinologie-Diabétologie, Paris, France; ^4^ Post Graduate School of Pediatrics, Departement of Medical and Surgical Sciences of the Mothers, Children and Adults, University of Modena and Reggio Emilia, Modena, Italy; ^5^ Pediatrics, IRCCS Casa Sollievo della Sofferenza, San Giovanni Rotondo, Italy; ^6^ PhD Student, University of Fogggia, Foggia, Italy; ^7^ French Clinical Research Group in Adolescent Medicine and Health, Paris, France

**Keywords:** iodine, iodine deficiency, pediatrics, adolescents, pregnancy, fetus

## Abstract

The aim of this mini-review is to present the current knowledge on iodine requirements in developmental age, from conception to adolescence. It is based on the analysis of updated national and international guidelines on iodine intake and the prevention of iodine deficiency. Health policy initiatives carried out in industrialized countries in previous decades have led to a dramatic improvement in nutritional iodine status in the general population. However, the prevention of iodine deficit continues to be a concern, especially for vulnerable categories, like adolescents and pregnant women.

## Introduction

Iodine is a non-metallic trace element present in minimal quantities in the body (15-20 mg total in adults), mostly concentrated in the thyroid. It plays an essential role since the first moment of conception, because it oversees the formation of thyroid hormones. Indeed, the embryo first and the fetus later on are completely dependent on maternal thyroid hormones in the first part of gestation. While the hypothesis that thyroxine (T4) does not cross the placenta and has no role in the first weeks of gestation is outdated, there is no doubt on the importance of avoiding iodine deficiency during pregnancy. In addition, iodine deficiency since the first months of life has significant repercussions on the psychosomatic development of children.

Health policies adopted in many countries to prevent iodine deficiency have led to a significant improvement in dietary iodine status. In fact, although in recent decades, we have witnessed a significant reduction in geographic areas with iodine deficiency, further efforts are needed for implementation strategies.

The first aim of this mini-review is to present the role of iodine in the formation of thyroid hormones, underlining their fundamental action in the neuronal and somatic development since fetal life.

Secondly, it will focus on the current knowledge providing the indications for correct iodine intake and prevention of iodine deficiency, starting from the principles of physiology. It will highlight the guidelines issued by national and international scientific societies, analyzing the methods of iodine intake assessment.

Furthermore, open question about specific diets, such as ovo-lacto vegetarianism and veganism, and the use of food supplements will be discussed.

## Synthesis of Thyroid Hormones

**Figure 1:**
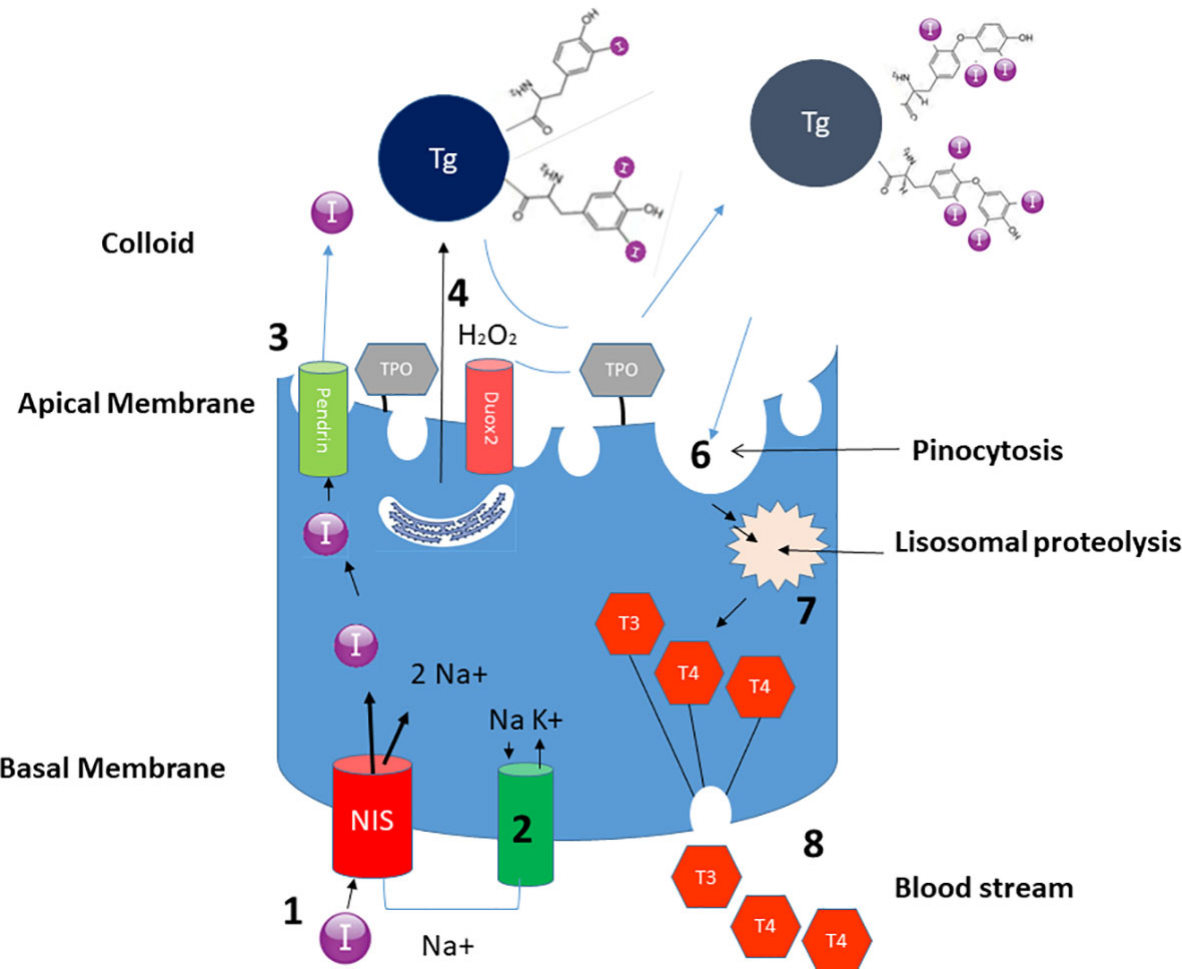
Schematic representation of the role of iodine in the biosynthesis of thyroid hormones. Thanks to the action of NIS, iodine is incorporated in the thyrocyte (1). The process is guaranteed by the Na+ electrochemical gradient generated by the Na+/K+ ATPase on the basolateral membrane (2). Iodine subsequently crosses the apical membrane carried by pendrine (3) – and probably also by other transporters, to reach the colloid. Thyroglobulin, synthesized in the rough endoplasmic reticulum, reaches the colloid through exocytosis (4). The iodine present in the colloid is oxidized and incorporated in thyroglobulin, through the enzymatic mechanism of TPO thanks to H2O2 generated by Duox2 (5) Iodized thyroglobulin is internalized with a mechanism of pinocytosis (6) and undergoes proteolysis (7) giving rise to the formation of T4 and T3 which are then conveyed to the bloodstream (8).

Iodine plays a key role in thyroid hormonogenesis. Thyroid hormone synthesis occurs in the follicles (**Figure 1**). Iodine travels in the blood stream as iodide (I^-^); the sodium-iodide cotransporter, also known as sodium-iodide symporter (NIS), accounts for the uptake and interiorization of iodide by the basolateral membrane of thyrocytes from the bloodstream. Thereafter, iodide reaches the colloid in the middle of follicles crossing the apical membrane carried by other cotransporters, such as pendrin. In the colloid, due to the activity of thyroid peroxidase (TPO) and the presence of hydrogen peroxide (H_2_O_2_), iodide is oxidized into iodine, and is incorporated in the iodotyrosine of thyroglobulin, leading to the formation of mono and diiodotyrosine (MIT and DIT): this process is called organification. Afterwards, TPO couples two iodotyrosines and incorporates them into thyroglobulin to create T4 or triiodothyronine (T3). When the follicular cells are stimulated by thyroid-stimulating hormone (TSH), and when there is need to release the hormones, thyrocytes incorporate thyroglobulin from colloid by endocytosis and micropinocytosis and after lysosomal digestion, T3 and T4 are released. Unused MITs and DITs are deiodated by iodothyrosine dehalogenase (DEHAL1), which releases iodine and recycles it for further hormone synthesis (1).

## The Intake and Requirement of Iodine

Under normal conditions, the dietary intake of iodine comes mainly from seafood (fish, crustaceans, mollusks), which may contain up to 400 µg/100g (**Table 1**) (2). Atmosphere iodine is scarcely available and practically has no influence on the calculation of needs.

**Table 1:** Average content of iodine in the most common foods (Italian National Observatory for the monitoring of iodine prophylaxis – OSNAMI).

Food category	Food	µg iodine/g of wet mass(M ± DS)
** Fish and fishery products**	Mussels, clams, squid, red mullet, cod, Atlantic tuna, European hake	0.74±0.24
** Cereals (pasta and bread)**	Pasta, pizza, breakfast cereals	0.06±0.02
** Milk and milk-based drinks**	Milk, yogurt	0.15±0.06
** Semi-matured cheese**	Cow cheese	0.30±0.06
** Meat**	Beef, pork, poultry	0.03±0.01
** Egg**	-	0.08± 0.03
** Fruit/vegetable**	Leafy vegetable, tomato, root, fresh fruit	0.03±0.01
** Water and drinks**	-	<cutoff detection
** Salt**	Coarse iodized salt, fine iodized salt	29.8±2.5

Ingested iodine is absorbed in the small intestine, but its persistence in blood circulation is relatively short, since the renal plasma clearance of iodide is particularly high (35 ml/min) compared to the clearance of chlorine, which is 1 ml/mil). This means that, although the percentage of absorption is 92%, within 72 hours two-thirds of the ingested iodine is excreted in urine and almost all the rest is conveyed to the thyroid (1, 3). These physiologic features are essential for two issues that will be addressed later: possible malabsorptive conditions that may induce iodine deficiency and the use of urinary output of iodine to define the nutritional status of this trace element.

## Levels of Adequate Intake: EFSA Guidelines

The European Food Society Authority (EFSA) proposes age-specific intakes of iodine ranging from 70µg/24h for children to 150µg/24h at the end of adolescence (4). These are intakes calculated to ensure a Urinary Iodine Concentration (UIC, see below) ≥ 100 µg/l, which is the cut-off associated with the lowest prevalence of iodine-deficient goiter in school-aged children (5).

### Once Deficit Has Been Avoided, Beware of Excess

EFSA has set an upper safety limit for adults at 600 µg/day. Moreover, a document issued by the Italian Society for Human Nutrition, (Società Italiana Nutrizione Umana, SINU) (5) recommends the maximum tolerable levels of iodine intakes for different age groups: in pediatric age it goes from 40µg/day in infants to 200µg/day up to age 3 years, and 500µg/day at the end of the adolescence. Pregnant and breastfeeding women have the same requirements as the rest of the adult population. The indication of the maximum tolerable levels of iodine is of utmost importance because an iodine excess can have deleterious effects at least as much as a deficit.

The thyroid has a defense mechanism to avoid iodine excess; indeed, in case of excessively high intakes, there is a decrease in thyroid sensitivity to TSH and an inhibition of iodine uptake, or even the inhibition of the secretion of thyroid hormones (Wolff-Chaikoff effect). This phenomenon is useful in the prevention of harmful effects in the event of nuclear accidents and in presence of radioactive clouds: the administration of potassium iodide tablets almost immediately block iodine uptake by the thyroid, avoiding the storage of radioactive elements in the gland (6).

In most cases, iodine excess is of iatrogenic origin (amiodarone, iodinated contrast agents), but sometimes can also be food-related or due to a poorly controlled composition and/or intake of food supplements (self-prescriptions and “do-it-yourself” diets, particularly fashionable in this social era). There is evidence that in the presence of excessive iodine intake, there is an increase in thyroid dysfunctions even among children, with a spectrum ranging from overt hypothyroidism to hypothyroidism, passing through subclinical hypothyroidism as well as specific autoimmunity phenomena (7, 8). A recent study carried out on a population of South-Korean children, aged 6-19 years old, found a correlation with subclinical hypothyroidism (SCH), defined as TSH > 5.5mUI/l, in individuals either with a condition of iodine-deficiency (UIC <100 µg/l) or iodine-excess (>300 µg/l) (9). Regardless of the definition of SCH – a not well-defined entity mipediatric age (10), the tendentially “U” curve drawn on the basis of TSH value variation as a function of ioduria showed how the effects of iodine deficit could be comparable to those of excess intake.

Moreover, it is interesting that in that study, SCH was present starting from a condition of “mild iodine excess” (i.e. UIC 300 – 599 µg/l), even before moderate (i.e. 600 – 999.9 µg/l) or severe (i.e. > 1000 µg/l) excess was reached.

Concerning the recommendations to the general population, it is important to underline that no additional supplementation is needed other than suggested by public health measures (salt or other iodized food). An Italian survey aiming to evaluate the correlation between salt consumption and iodine intake in a pediatric population showed that 24/1888 children had been excluded from the evaluation because they had ioduria >400 µg/l, probably due to the intake of iodine-rich food or to the absorption not of dietary origin (local antiseptics, sanitizers or toothpaste containing iodine) (11).

Special attention should be reserved to food supplements sold and often seen as “neutral” product, wrongly perceived as harmless.

## The Requirement of Iodine in Different Stages of Life

### Fetal Life: The Necessary Amount of Iodine During Pregnancy

Maternal T4 crosses the placenta. Therefore, even if the circulating T4 levels in the bloodstream of the fetus are 100 times lower than those detected in the mother, free T4 (fT4) reaches adult levels and T3 reaches adult levels in the cerebral fetal cortex thanks to the local deiodinases. The maternal thyroid provides thyroid hormones to fetal tissues before the thyroid gland of fetus begins to function. At birth, maternal T4 represents about 20-50% of the T4 concentration in the cord blood. For all these reasons, maternal iodine status is critical for embryonic and fetal development. To correctly evaluate the maternal thyroid function during gestation, it is important to know that in the early stages of pregnancy some women may have low TSH levels. This is due to the fact that human chorionic gonadotropin (hCG) is a glycoproteic hormone belonging to the same family of TSH and therefore may bind the TSH receptor causing an increase in thyroid hormone (12).

Pregnant women risk iodine deficiency because of the significantly higher requirements (about 250 µg vs 150 µg/day) in this phase.

Different studies, such as those from neonatal cohorts from England (ALSPAC), Spain (INMA) and The Netherlands (Generation R), showed that iodine supplementation in the preconception period, and in any case before the first trimester of pregnancy, ensured the best results (13). One can argue that this is a confirmation of how important it is to avoid or correct any deficiencies (i.e., folic acid) before conception rather than after the diagnosis of pregnancy. However, the systematic prescription of iodine supplement in pregnant women living in areas not classified as iodine deficient is not unanimously accepted. In 2019 the Iodine Global Network Annual Report detected only 23 nations as iodine deficiency areas (3 in Europe: Finland, Norway, Germany), instead of the 54 listed in 2003 and 113 in 1993. Even if it is undeniable that severe iodine deficiency during gestation has a negative impact on the psychodevelopmental outcome, the effect of a mild or moderate deficit is controversial. A recent meta-analysis failed to show sufficient data in favor of iodine supplementation in pregnant women living in areas with mild or moderate deficiency (14), arousing current ideas for reflections (15).

### The Need for Iodine During Breastfeeding

Milk plays a key role in the iodine supply for infants, with an average iodine content of 259 µg/l (with great disparities: from 79 µg/l to a maximum of 400 µg/l). The change in iodine content in milk results from seasonality and supplementation of iodine in animal feed, while there are no differences between fresh and long-life milk or between whole and skimmed milk. For this reason, the use of iodized salt in food is not recommended as long as milk remains the predominant food in a baby’s diet. A 4-months-old baby will take on average 117 µg/day of iodine with milk formula. On the other hand, as iodine in breastmilk depends on maternal iodine status, the iodine intake in a breastfeeding mother needs to be around 200 µg/day (the same requirement needed in pregnancy).

### Children and Adolescents

Once the adequate iodine intake has been ensured (EFSA aims to achieve a ioduria >100 µg/l, value related to a minimum incidence of goiter in the growing population), particular attention must be paid to nutritional intake, especially nowadays when gastronomic influences from all over the world have substantially changed many of the eating habits of adolescents. In a group of Italian children and adolescents (11), the iodine requirement was satisfied thanks to an excessive intake of salt (>10.2 g/day). This data highlighted not only an excessive salt intake but also that most iodine intake comes from iodized salt, which is still used in insufficient manner in industrial processes and by catering services. In the same study (11), an estimated value of iodine intake from unsupplemented food in different age groups was estimated as 44 µg/1000 Kcal for children, 45µg/1000Kcal for teenage girls and 35µg/1000Kcal for teenage boys. Additionally, it is a widespread concept that some foods, such as vegetables belonging to the Brassicaceae family, cassava, and millet, play a role in iodine malabsorption, although this is true only when these foods are consumed in large quantities and in a condition characterized by iodine deficiency. However, it is interesting to focus on the mechanism of action mediated by thiocyanates, which have a competitive action on the uptake of iodine by thyrocytes, and promote the efflux of intrathyroidal iodine. Thiocyanates are present in significant amounts in cigarette smoke, which explains the association between smoking and goiter, with an even greater effect in obesity (16). This could be another reason to reinforce prevention campaigns against smoking and obesity in this age group.

### The Vegetarianism

The proliferation of “particular” diet regimens laid down by cultural, ideological, or belief-based dictates of alternative medicine can be at the origin of an insufficient contribution of iodine. Vegetarianism (lacto-ovo vegetarianism and veganism) has the merit of being codified and of paying much attention to food supplements. A recent European survey showed how nutritional intakes were met in vegetarian children and adolescents (17). Moreover, SINU has approved the use of this diet also in pregnancy and breastfeeding (18). In the literature there are some case reports of severe iodine deficiency resulting, however, from a personal – and extreme – interpretation of the vegetarian diet taken, characterized by the refusal of food supplements and iodized salt (19).

In conclusion, if parents do not take personal initiatives and accept to give food supplements to their children, a selective diet, such as vegetarianism, does not pose any kind of problem.

### Public Health Policies: The Iodized Salt

An essential role is played by the intake of iodized salt; indeed, this is a health policy issue in many countries. In Italy, iodine supplementation with salt (which contains on average 30 µg/g compared to sea salt which contains 16 µg/g) is regulated by law (law n. 55 issued on March 21^st^, 2005), as part of the provisions aimed to the prevention of endemic goiter and other pathologies due to iodine deficiency.

One of the current issues is whether the advice to optimize iodine intake though salt is compatible with the need to reduce salt intake in the whole population, including the pediatric one. In fact, the maximum intake recommended by WHO for the prevention of cardiovascular diseases should not exceed 5 g/day in adults. This intake corresponds to 150 µg/day of iodine (for iodized salt), enough for most age groups but insufficient during pregnancy and breastfeeding. Moreover, 5 g of salt is the maximum intake recommended, although it is suggested that it be reduced to 3 g (90 µg of iodine). This leads to the question of “How to reduce salt intake and have a correct iodine supplementation ? »

We have to consider that iodized salt is not the only source of iodine in the diet. A portion of iodine is supplied by food and by water drunk or used for cooking. This quantity can be different depending on individual food choices and on the iodine content in the water, which varies from country to country, but still represents a signifcant amount of the total intake. Hence, the iodine provided by the iodized salt is an integration, almost always necessary, but which completes the amount of iodine already taken by other means. In other words, iodine in iodized salt is not the only contribution of this element; in a low sodium diet, it is possible to decrease the amount of iodized salt added without experiencing an iodine deficiency.

Therefore, it remains important to ensure the remaining intake with the food present in a varied diet, refecting as much as possible a Mediterranean type of diet (11, 20).

It is also fundamental to point out that the so-called “iodine allergy” is a misnomer used in case of reactions to iodine-based contrast; it does not correspond to a real allergy to the trace element but to an idiosyncrasy to one of the excipients used in the contrast means. The use of iodized salt in these patients is not contraindicated.

## The Evaluation of the Iodine State

### Population Studies

Most of the iodine absorbed is eliminated by the kidneys; for this reason, the Urinary Iodine Excretion (UIE) is the most sensitive parameter to evaluate recent iodine intake and, consequently, it is a good indicator of the iodine status of a population. UIE is expressed as µg/24h; other ways to assess iodine concentrations in urine are the Urinary Iodine Concentration (UIC) – corresponding to the quantity of iodine in a liter of urine (µg/l), and the assessment of urinary creatinine (UIC(µg/l) x 0.0235 x weight in kg).

The 24-hours urinary collection is often found to be a complex method and therefore not very reliable (21). To simplify, in most studies a single urinary sample is used, usually expressing the result as µg/l (or µg/g of creatinine). Even if this method is more feasible, it could be inadequate especially in children; since the value is related to a volume of 1 liter, in a child with a physiologically lower urinary secretion, the result obtained would be overestimated. For example: an excretion of iodine of 50 µg in a day with a urinary volume of 500 ml expressed in µg/l as 100 µg/l, would provide an incorrect interpretation of the result; in fact, if 50 µg were the entire quote(amount) of iodine excreted in 24 hours, it would result to be less than the lower limit of UIE (corresponding to 70µg) recommended by EFSA in this age group.

This approximation of the single urinary sample expressed in µg/l can be used when the urinary excretion of the individual is near to 1l/day, and for the pediatric age this can be applied in older children and adolescents (22–24).

## Conclusions

Iodine is a fundamental trace element in every stage of life, from conception to adulthood. Health policies aimed at preventing iodine deficiency have allowed a marked improvement in the iodine status of the Italian population. Particular attention should be paid to the most vulnerable populations (pregnant women and adolescents) in whom the higher iodine requirements may not be completely satisfied.

Future preventive strategies of iodine deficiency should promote the consumption of food naturally rich in iodine (milk and diary, fish), even before the hypothesis of increasing the concentration of iodine in salt, because this could expose part of the pediatric and adolescent population, the most virtuous on nutrition, to an excess salt intake.

## Author Contributions

GDF, AC and DR conceptualized and designed the review, drafted the initial manuscript and approved the final manuscript as submitted. GI conceptualized the review, performed a detailed bibliographic analysis, drafted the initial manuscript and approved the final manuscript as submitted. VT performed a detailed bibliographic analysis, critically reviewed the manuscript and approved the final manuscript as submitted. IR and VA critically reviewed the manuscript, updated bibliography and approved the final manuscript as submitted. All authors have read and agreed to the published version of the manuscript.

## Conflict of Interest

The authors declare that the research was conducted in the absence of any commercial or financial relationships that could be construed as a potential conflict of interest.

## Publisher’s Note

All claims expressed in this article are solely those of the authors and do not necessarily represent those of their affiliated organizations, or those of the publisher, the editors and the reviewers. Any product that may be evaluated in this article, or claim that may be made by its manufacturer, is not guaranteed or endorsed by the publisher.

## Abbreviations

NIS, sodium/iodine cotransporter; Tg, thyroglobulin; TPO, thyroperoxidase; T4, Thyroxine; T3l, triiodotironine.

## References

[1] Portulano C Paroder-Belenitsky M Carrasco N. The Na+/I- Symporter (NIS): Mechanism and Medical Impact. Endocr Rev (2014) 35(1):106–49. doi: 10.1210/er.2012-1036 PMC3895864 24311738

[2] Pastorelli AA Stacchini P Olivieri A. Daily Iodine Intake and the Impact of Salt Reduction on Iodine Prophylaxis in the Italian Population. Eur J Clin Nutr (2015) 69(2):211–5. doi: 10.1038/ejcn.2014.206 25293434

[3] Nicola JP Basquin C Portulano C Reyna-Neyra A Paroder M Carrasco N. The Na+/I- Symporter Mediates Active Iodide Uptake in the Intestine. Am J Physiol Cell Physiol (2009) 296(4):C654–62. doi: 10.1152/ajpcell.00509.2008 PMC2670652 19052257

[4] European Food Safety Authority Panel 2014. Scientific Opinion on Dietary Reference Values for Iodine. EFSA J (2014) 12:3660. doi: 10.2903/j.efsa.2014.3660 PMC13159826 42125360

[5] Delange F Benker G Caron P Eber O Ott W Peter F. Thyroid Volume and Urinary Iodine in European Schoolchildren: Standardization of Values for Assessment of Iodine Deficiency. Eur J Endocrinol (1997) 136(2):180–7. doi: 10.1530/eje.0.1360180 9116913

[6] Campagne De Distribution D’iode. Available at: https://www.interieur.gouv.fr/Le-ministere/Securite-civile/Nos-missions/La-protection-des-personnes-des-biens-et-de-l-environnement/Campagne-de-distribution-d-iode (Accessed 25/10/2021).

[7] Sang Z Chen W Shen J Tan L Zhao N Liu H. Long-Term Exposure to Excessive Iodine From Water Is Associated With Thyroid Dysfunction in Children. J Nutr (2013) 143(12):2038–43. doi: 10.3945/jn.113.179135 24108132

[8] Chung HR Shin CH Yang SW Choi CW Kim BI. Subclinical Hypothyroidism in Korean Preterm Infants Associated With High Levels of Iodine in Breast Milk. J Clin Endocrinol Metab (2009) 94(11):4444–7. doi: 10.1210/jc.2009-0632 19808851

[9] Kang MJ Hwang IT Chung HR. Excessive Iodine Intake and Subclinical Hypothyroidism in Children and Adolescents Aged 6-19 Years: Results of the Sixth Korean National Health and Nutrition Examination Survey, 2013-2015. Thyroid. (2018) 28(6):773–9. doi: 10.1089/thy.2017.0507 29737233

[10] Salerno M Capalbo D Cerbone M De Luca F. Subclinical Hypothyroidism in Childhood - Current Knowledge and Open Issues. Nat Rev Endocrinol (2016) 12(12):734–46. doi: 10.1038/nrendo.2016.100 27364598

[11] Iacone R Iaccarino Idelson P Campanozzi A Rutigliano I Russo O Formisano P. Relationship Between Salt Consumption and Iodine Intake in a Pediatric Population. Eur J Nutr (2021) 60(4):2193–202. doi: 10.1007/s00394-020-02407-w PMC8137629 33084957

[12] de Escobar GM Obregón MJ del Rey FE. Maternal Thyroid Hormones Early in Pregnancy and Fetal Brain Development. Best Pract Res Clin Endocrinol Metab (2004) 18(2):225–48. doi: 10.1016/j.beem.2004.03.012 15157838

[13] Levie D Korevaar TIM Bath SC Murcia M Dineva M Llop S. Association of Maternal Iodine Status With Child IQ: A Meta-Analysis of Individual Participant Data. J Clin Endocrinol Metab (2019) 104(12):5957–67. doi: 10.1210/jc.2018-02559 PMC6804415 30920622

[14] Dineva M Fishpool H Rayman MP Mendis J Bath SC. Systematic Review and Meta-Analysis of the Effects of Iodine Supplementation on Thyroid Function and Child Neurodevelopment in Mildly-to-Moderately Iodine-Deficient Pregnant Women. Am J Clin Nutr (2020) 112(2):389–412. doi: 10.1093/ajcn/nqaa071 32320029

[15] Ley D Turck D. Iodine Supplementation: Is There a Need? Curr Opin Clin Nutr Metab Care (2021) 24(3):265–70. doi: 10.1097/MCO.0000000000000737 33587366

[16] Rendina D De Palma D De Filippo G De Pascale F Muscariello R Ippolito R. Prevalence of Simple Nodular Goiter and Hashimoto's Thyroiditis in Current, Previous, and Never Smokers in a Geographical Area With Mild Iodine Deficiency. Horm Metab Res (2015) 47(3):214–9. doi: 10.1055/s-0034-1387702 25153684

[17] Sobiecki JG Appleby PN Bradbury KE Key TJ. High Compliance With Dietary Recommendations in a Cohort of Meat Eaters, Fish Eaters, Vegetarians, and Vegans: Results From the European Prospective Investigation Into Cancer and Nutrition-Oxford Study. Nutr Res (2016) 36(5):464–77. doi: 10.1016/j.nutres.2015.12.016 PMC4844163 27101764

[18] Documento SINU Sulla Dieta Vegetariana . Available at: https://sinu.it/wp-content/uploads/2019/06/documento-diete-veg-esteso-finale-2018.pdf (Accessed 25/10/2021).

[19] Yeliosof O Silverman LA. Veganism as a Cause of Iodine Deficient Hypothyroidism. J Pediatr Endocrinol Metab (2018) 31(1):91–4. doi: 10.1515/jpem-2017-0082 29303778

[20] Rutigliano I De Filippo G Campanozzi A. The Importance of Reducing Salt Intake in Children, While Respecting the Correct Iodine Supplementation. The Pediatricians' Point of View. High Blood Press Cardiovasc Prev (2020) 27(6):601–2. doi: 10.1007/s40292-020-00413-x 33010008

[21] König F Andersson M Hotz K Aeberli I Zimmermann MB. Ten Repeat Collections for Urinary Iodine From Spot Samples or 24-Hour Samples Are Needed to Reliably Estimate Individual Iodine Status in Women. J Nutr (2011) 141(11):2049–54. doi: 10.3945/jn.111.144071 21918061

[22] Zimmermann MB Andersson M. Assessment of Iodine Nutrition in Populations: Past, Present, and Future. Nutr Rev (2012) 70(10):553–70. doi: 10.1111/j.1753-4887.2012.00528.x 23035804

[23] Chen W Wu Y Lin L Tan L Shen J Pearce EN. 24-Hour Urine Samples Are More Reproducible Than Spot Urine Samples for Evaluation of Iodine Status in School-Age Children. J Nutr (2016) 146(1):142–6. doi: 10.3945/jn.115.215806 26609173

[24] Campanozzi A Rutigliano I Macchia PE De Filippo G Barbato A Iacone R. Iodine Deficiency Among Italian Children and Adolescents Assessed Through 24-Hour Urinary Iodine Excretion. Am J Clin Nutr (2019) 109(4):1080–7. doi: 10.1093/ajcn/nqy393 30982855

